# Non-Destructive Genotyping of Honeybee Queens to Support Selection and Breeding

**DOI:** 10.3390/insects11120896

**Published:** 2020-12-21

**Authors:** Jernej Bubnič, Katarina Mole, Janez Prešern, Ajda Moškrič

**Affiliations:** Animal Production Department, Agricultural Institute of Slovenia, Hacquetova ulica 17, SI1000 Ljubljana, Slovenia; jernej.bubnic@kis.si (J.B.); mole.katarina@gmail.com (K.M.); janez.presern@kis.si (J.P.)

**Keywords:** *Apis mellifera carnica*, Carniolan honeybee, DNA extraction, exuviae, feces, honeybee queen, genotyping, non-destructive DNA sampling, dCAPS markers, hygienic behavior

## Abstract

**Simple Summary:**

Genotyping of an individual usually requires tissue samples obtained by methods that may cause an injury. In the case of honeybee queens, most of such injuries cause supersedure of the queen. We have investigated the possibility of using two non-destructive sources to obtain genetic information from an individual queen: feces and exuviae. Both sources turned out to be acceptable in terms of quality of DNA and usefulness in genotyping. In practical use, any of the two could be used to shorten generation interval in queen breeding and decrease costs of the selection process.

**Abstract:**

In traditional bee breeding, the honeybee queen is chosen for breeding based on the performance of the colony produced by its mother. However, we cannot be entirely certain that a specific queen will produce offspring with desirable traits until we observe the young queen’s new colony. Collecting the queen’s genetic material enables quick and reliable determination of the relevant information. We sampled exuviae, feces, and wingtips for DNA extraction to avoid fatally injuring the queen when using tissue samples. Quantity and purity of extracted DNA were measured. Two mitochondrial markers were used to determine the lineage affiliation and exclude possible contamination of DNA extracts with non-honeybee DNA. dCAPS (derived Cleaved Amplified Polymorphic Sequences) markers allowed detection of single nucleotide polymorphisms (SNPs) in nuclear DNA regions presumably associated with Varroa sensitive hygiene and set the example of successful development of genotyping protocol from non-destructive DNA sources. One of the logical future steps in honeybee breeding is introducing genomic selection and non-destructive sampling methods of genetic material may be the prerequisite for successful genotyping. Our results demonstrate that the extraction of DNA from feces and exuviae can be introduced into practice. The advantage of these two sources over wingtips is reducing the time window for processing the samples, thus enabling genotyping directly after the queen’s emergence.

## 1. Introduction

Genotyping and marker-assisted selection (MAS) are considered as a necessary step in the process of selection and breeding of honeybee queens, citing health-related reasons [1,2] linked to the economic success of the beekeeping operations [3]. Recent advances in the field of genomics provide insights also into the genetic background of economically important traits [4] as well as into the lineage and population-specific signatures [5], often vital for conservation [6].

The first and arguably crucial step for successful genotyping is to extract the necessary amount of DNA of required purity and quantity from the biological sample. The extraction of adequate DNA from insect tissues for various purposes, honeybees being no exception, is considered routine [7,8]. The drawback of the majority of these approaches is the necessity of sacrificing the individual or damaging it to acquire tissue samples for further analysis.

In honeybee queens, for example, such an approach can be especially problematic; even modest injury such as a damaged leg might lead to the replacement of the queen by workers in the colony [9,10,11]. In case of honeybees, the general quality and genome properties of the queens of a certain pedigree line can be evaluated through their relatives, namely their sons (drones). The queens’ genotype representation in drone brood has been studied by sampling drone eggs and compared the detected loci to the queen’s own with DNA obtained from the muscle tissue. Drone eggs as an alternative source were shown to be reliable [12], and even with thirty drone eggs, the difference in the call between queen muscle tissue and pooled eggs was 0.17% on average. A drawback is a “refractory” period before the queen’s offspring may be sampled and thus, immediate genotyping after the virgin queen emerges is not possible [12]. This also increases the cost of operation by forming nucleus colonies to sustain them until genotyping via drones is possible.

The alternatives to the destructive approach are semi- or non-destructive sampling techniques. A widely used semi-destructive technique is routine wing clipping, usually done by beekeepers to mark the queen’s age and partially limit swarming. The clipped wing is considered less detrimental to the well-being of the queen. The successful extraction of DNA from clipped wings and subsequent genotyping were reported in the past [13,14,15,16,17]. Recent research demonstrated the efficient high throughput method to identify *csd* alleles of the honeybee queens using wing clippings coupled with a next-generation sequencing approach [18]. Though successful, there is still a requirement to wait with wing clipping until mating flights are completed. 

Non-destructive samples that arise directly from the queen include chitin exuviae, the remains in the queen cell after the emergence. The cuticle itself does not contain cells; however, some cells are attached to the molt parts belonging to the anterior and posterior gut and trachea [19], enough for successful extraction of genetic material and subsequent genotyping. The success in the extraction of DNA from exuviae was demonstrated in some insect groups [20,21] including honeybees [14]. Their work laid the foundation for an early genotyping of queens, assuring that only those queens possessing specific traits identified molecularly will be placed in a colony [14]. Most recently, queen exuviae were also successfully used as a starting material for genotyping honeybee queens using commercially available honeybee ChipSNP [12]. In another study, the patriline composition of honeybee workers exhibiting fanning behavior was determined using exuviae as a non-destructive genotyping source [22], demonstrating the utility of such starting material for observing behavioral and genotypic dynamics in natural honeybee colonies.

The other promising non-destructive source of DNA is feces. Although the collection of the feces of honeybee queens is described in [8], our study is the first to demonstrate the successful utility of the feces of honeybee queens for non-destructive genotyping. A similar method was demonstrated to study other hymenopterans’ population genetics, such as bumblebees [23]. Insect feces were also used to detect insect taxa that are considered plant pathogens [24] and in investigations of other insect–host plant interactions [25]. A recent addition to the implementation of the Slovenian domestic queen breeding program [26] is sampling the queen feces for genotyping when queens are being collected for distribution among performance testers, thus making it possible to determine certain genetic parameters before the testing starts.

The goal of our study was to investigate the possibilities for successful extraction of honeybee queens’ DNA from source obtained through non-destructive sampling methods allowing classical genotyping of the queen before it “takes over” the colony. Here we report on the successful extraction of DNA using two non-destructive sampling methods, both simple and efficient for further amplification and genotyping. Two mitochondrial markers were used to obtain information important for determining Western honeybee lineages and contamination monitoring. Using dCAPS (derived Cleaved Amplified Polymorphic Sequences) protocol to detect selected single nucleotide polymorphisms (SNPs) in nuclear DNA, we demonstrated the possible genotyping method targeting specific genotypic traits in aid of future breeding programs. A simple step-by-step protocol presented in the methodological part of the paper can be forwarded to queen breeders to properly process queen cells after the queen’s emergence for genotyping purposes.

## 2. Materials and Methods

### 2.1. Samples Information

Samples in this study consisted of several tissue types from honeybee queens (legs, antennae, wing clippings) and workers (legs) and two non-destructive queen sources (exuviae in queen cell after the emergence of the virgin queen, and queen’s feces). All the different mitochondrial loci variants were deposited in the Genbank repository (NCBI—https://www.ncbi.nlm.nih.gov/genbank/) and are presented in Supplementary Materials—Table S1.

### 2.2. Feces Sampling

Samples of honeybee queens’ feces were collected following recommendations [8] with some modifications. Briefly, each honeybee queen was anesthetized using CO_2_ gas and transferred from the queen cage to a clean and sterile Petri dish, placed on the white paper sheet. The queen usually defecated within minutes after waking up. The queen was immediately removed from the Petri dish and feces was dissolved in 200 µL of sterile bi-distilled water. The solution was then transferred to a sterile microcentrifuge tube and stored at −20 °C until DNA extraction. Feces samples of honeybee queens were collected when queens were being prepared for distribution to beekeepers for performance testing in 2019. Altogether 16 samples of honeybee queens’ feces were collected from 5 different breeders.

### 2.3. Sampling of Exuviae

**Figure 1:**
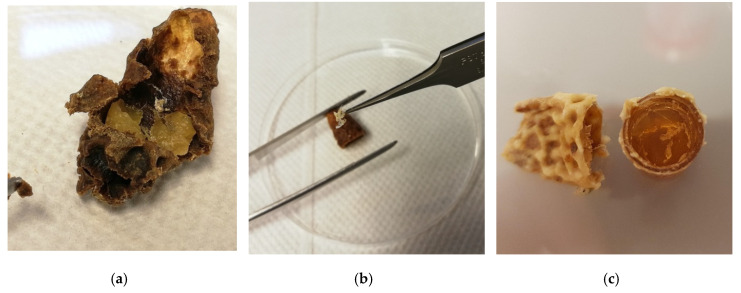
Examples of queen cell after the queen’s emergence and sampling of exuvia: (**a**) an opened queen cell. The yellowish mass at the bottom half is the remnant of royal jelly, while the tiny paper-like flake in the middle of the cell is the remnant of the exuvia. (**b**) removal of exuvia from the queen cell. (**c**) remnants of exuvia at the queen cell cup.

In cooperation with Slovenian queen breeders, we collected 198 queen cells from 14 queen breeders and beekeepers in 2019. We obtained queen cells immediately after the emergence of a virgin queen. We prepared a simple protocol for collecting, storing, and transporting the queen cells via regular postal service. Briefly, sterile pre-labeled 50 mL tubes were distributed to breeders. Breeders removed the queen cells immediately after the queens’ emergence, stored one queen cell per tube, and returned them in a 48 h window after emergence. The breeder equipped the tube with the date of emerging, the mother queen’s pedigree, and mating hive number if available. The collected samples were then stored at −20 °C until DNA extraction. If queens emerged in the colony, we have impressed upon breeders to remove queen cells as soon as possible before the workers would have the opportunity to clean them. Of the obtained queen cells, 135 contained chitin remnants sufficient to proceed with the extraction. Using sterile spatula and forceps, we have removed the paper-like, white to yellow structures (remnants of cuticle after the last molt of the queen; [19,27], Figure 1) deposited on the inner cell walls and transferred them into fresh 2 mL tube. In some cells, there was a yellowish mass present at the bottom, which was collected separately to extract DNA.

### 2.4. Tissue Sampling

Using sterile forceps, tissue samples such as legs, antennae, and wing clippings were obtained from queens, replaced in colonies in Institutes’ apiaries. Collected samples were stored in sterile microcentrifuge tubes at −20 °C, until extraction of DNA. Additionally, the legs of honeybee workers and drones from colonies with detected various hygienic behavior rates were collected from fresh or frozen specimens. Altogether, 61 samples (48 legs, 9 wing clippings, and 4 antennae) were collected for DNA extraction.

### 2.5. DNA Extraction

All DNA extractions were performed using the QiaAmp DNA Mini Kit (Qiagen, Germantown, MD, USA). Some of the parts of the extraction protocol were modified. Briefly, all samples except feces (collected exuviae, legs, antennae, and wing clippings) were homogenized in 80 µL of PBS buffer with two beads using TissueLyser (Qiagen) for one minute at 200 Hz. Next, 100 µL of ATL buffer and 20 µL of Proteinase K was added to each sample. After thorough mixing, the samples were incubated overnight at 56 °C. The following day 200 µL of AL buffer was added. The samples were mixed thoroughly again and incubated at 70 °C for 10 min. Afterwards, the samples were centrifuged at full speed for one minute and the supernatant was transferred into a clean microcentrifuge tube. Then the proportional volume of cold absolute ethanol was added, samples were thoroughly mixed and transferred into the spin columns. The liquid passed through by centrifugation (at 8000 RPM for 1 min) and was discarded. The spin-column was then washed twice. The first wash with 500 µL of AW1 buffer (8000 RPM for 1 min) was followed by a second wash with 500 µL of AW2 buffer (14,000 RPM for 3 min). Next, DNA was eluted from the membrane with AE buffer into a clean microcentrifuge tube. The elution volume depended on the starting material—DNA from exuviae was eluted in 50 µL, while DNA from tissue samples was eluted in 100 µL of AE buffer. DNA from the feces was extracted following the manufacturer’s “Protocol for DNA Purification from Blood or Body Fluids”. After the addition of ATL buffer and Proteinase K, the samples were incubated overnight at 56 ℃. All further steps followed the manufacturer’s protocol. DNA was eluted from the membrane with 50 µL of the AE buffer into a clean microcentrifuge tube. Quantity and purity of the extracted DNA were measured using BioSpectrometer (Eppendorf, Hamburg, Germany).

### 2.6. PCR Amplification, Purification, and Sequencing of Specific Fragments

Two mitochondrial markers were selected for amplification: tRNA^leu^-COX2 and cytochrome oxidase I (COI). Oligonucleotide primers and amplification protocol for each marker followed the literature [28,29]. Initial PCR reaction mixture volume was 15 µL and contained 7.5 µL of 2× DreamTaq MasterMix (Thermo Fisher Scientific, Waltham, MA, USA), 0.2 µL of each primer, 2 µL of extracted DNA and 5.1 µL of ddH_2_0 (Merck KGaA, Darmstadt, Germany). DNA extracts from legs were diluted 10× in ddH_2_O. Amplification was performed in thermocyclers SureCycler 8800 (Agilent, Santa Clara, USA), Veriti (Applied BioSystems, Thermo Fisher Scientific, USA), or T1 Thermocycler (Biometra, Bio-Sciences Limited, Dublin, Ireland).

Quality of amplification was verified by loading 5 µL of each amplified product on 1% agarose gel stained with ethidium bromide in 0.5× TBE buffer on horizontal electrophoresis. Products were visualized using a UV transilluminator at 280 nm. The length of fragments was estimated by comparing them to the standard GeneRuler 100 bp DNA (Thermo Fisher Scientific, USA), loaded simultaneously with the PCR products on one or more lanes. Following the manufacturer’s manual, selected PCR products were purified using ExoSAP-IT (Thermo Fisher Scientific, USA). Sequences were determined via the Sanger method using both sequencing primers at SeqMe (Dobříš, Czech Republic).

DNA chromatograms were assembled in Geneious Prime (https://geneious.com; Biomatters, Ltd., Auckland, New Zealand). The homologous sequences were aligned with ClustalW plugin [30] or, alternatively, with MAFFT v.7 plugin with the E-ins-I algorithm [31], both in Geneious Prime. All alignments were checked by eye for potential misaligned positions. COI sequences were translated into amino acid sequences and verified that they do not contain stop-codons. All obtained variants were verified using BLAST (https://blast.ncibi.nlm.nih.gov/Blast.cgi) and checked for homology with known sequences from the genus *Apis*. COI fragment, heavily utilized in the Barcoding organism identification method, and was verified using the BOLD database (https://boldsystems.org/index.php/IDS_IdentificationRequest).

### 2.7. Phylogenetic Analysis

Phylogenetic analysis of selected sequences from non-destructive, semi-destructive, and destructive sources was performed for tRNA^leu^-COX2 mitochondrial marker. Additional homologous sequences of the genus *Apis* were downloaded from GenBank (NCBI, https://www.ncbi.nlm.nih.gov) and included in the analyses. Eastern honeybee (*Apis cerana*—Genbank record DQ385854) was used as an outgroup and served to root the phylogenetic tree. Gaps in sequences were encoded as »-« and missing parts as »?«. The most appropriate substitution model under corrected Akaike Information Criteria (cAIC) was determined using jMODELTEST2 [32,33] in the CIPRES Science Gateway v3.3 [34].

The phylogenetic tree was calculated with MrBayes v.3.2.3 [35] at CIPRES Science Gateway by setting two parallel MCMCMC algorithms, with three hot and one cold chain each. For the tRNA^leu^-COX2 marker, the most suitable substitution model was HKY+G. The number of generations was adjusted to allow both algorithms to converge with a standard deviation of 0.01 or less; 2 × 10^6^ generations were enough to reach convergence. MrBayes sampled each 1000th generation. First, 25% of sampled trees were discarded as burn-in; the rest served to determine a 50% majority-rule consensus tree. Posterior probabilities showed statistical support at each node. The tree was visualized using FigTree 1.4.3 (https://tree.bio.ed.ac.uk/) and annotated for publication. 

### 2.8. Genotyping Using dCAPS Protocol

dCAPS markers were constructed to allow detection of single nucleotide polymorphism without the sequencing step. Several loci that contain SNPs reported to be associated with varroa sensitive hygiene, more specifically by detection and uncapping behavior of worker bees [2,36,37] were screened. From six of the SNPs with a significant genome-wide association with the trait “detection and uncapping of Varroa-infested brood cells” [2], the two of them were selected to develop further and optimize the genotyping protocol (Table 1). This selection’s prime criterion was constructing dCAPS primer pairs that enable efficient amplification of SNP containing region and recognition of certain variable site by the restriction enzyme.

For each locus, sequences flanking the polymorphic sites were imported to Geneious Prime. dCAPS Finder, 2.0 [38] on the server http://helix.wustl.edu/dcaps/ was used for the construction of oligonucleotide primer pairs for each SNP that enable detection of single polymorphic site/mutation using restriction with an endonuclease. The number of mismatches in the primer was set to 1. After selecting the derived oligonucleotide primer, its pair was constructed using Primer 3 v2.3.7 plug-in in Geneious Prime. Amplified fragments for each SNP-containing-region were around 200 bp long. Specific recognition of single nucleotide polymorphism by restriction endonuclease allowed the detection of different size fragments on agarose electrophoresis gel (Table 2).

For optimizing PCR conditions and confirmation of sequence specificity, each SNP-containing-fragment was first amplified from seven DNA extracts. All the conditions except for the annealing temperature were the same, as follows: initial denaturation step of 3 min at 94 °C was followed by 35 cycles of denaturation step of 15 s at 94 °C, annealing step of 30 s at various temperature (Table 2) and extension step of 30 s at 72 °C. The final extension step lasted for 5 min at 72 °C. The annealing temperature, length of the fragment, and lengths of restricted fragments for each SNP-containing-region are presented in Table 2. The sequences are presented in Supplementary Material—Figures S5 and S6.

Cleaning of PCR products, sequencing, and chromatogram editing procedures were the same as described above. Successfully read sequences were aligned and *in silico* checked for restriction endonuclease recognition site. The identities of the obtained sequences were confirmed using BLAST (httsp://blast.ncibi.nlm.nih.gov/Blast.cgi). After the optimization of PCR, the digestion with selected restriction endonuclease was performed. For SNP2 using restriction endonuclease *Alu*I (Thermo Fisher Scientific, USA), the restriction mixture contained 0.3 µL of restriction enzyme, 2 µL of 10× Tango Buffer, 2.7 µL ddH_2_O (Sigma, USA), and 15 µL of PCR product. For SNP3 using restriction endonuclease *Mnl*I (NewEngland Biolabs, Ipswich, MA, USA), the restriction mixture contained 0.6 µL of restriction enzyme, 1.4 µL of CutSmart buffer, 8 µL ddH_2_O, and 10 µL of PCR product. Both restriction mixtures were incubated overnight at 37 °C. Cleaved PCR products were visualized on 3% agarose gel electrophoresis in 0.5× TBE buffer stained with ethidium bromide. The length of cleaved fragments was determined by comparison to GeneRuler 100 bp Plus DNA Ladder and O’RangeRuler 10 bp DNA Ladder, ready-to-use (Thermo Fisher Scientific, USA)

## 3. Results

### 3.1. Sampling from Non-Destructive Sources and Extraction of DNA

To explore the possibilities of non-destructive genotyping of individual honeybee queens before their mating and creation of dedicated nucs/colonies for that purpose, we obtained DNA samples by using two non-destructive approaches (sampling of exuviae of queen cells after emergence and queens’ feces). Results were compared against samples obtained by semi-destructive and destructive methods. Of the collected 198 queen cells, a total of 135 contained sufficient exuvial remnants to be further processed. A yellowish mass that was present in some of the queen cells was found to be a less suitable starting material for this type of extraction method—after the lysis, the remains of the mass does not form a separated precipitate, and the membrane of the column is easily clogged in subsequent washing steps; thus, successful extraction of DNA is compromised. On the contrary, paper-like chitin remnant usage as a starting DNA source did not present any complications during the extraction.

Concentrations of DNA obtained from non-destructive sources (feces and exuviae) were low and of suboptimal purity. Only the legs provided higher DNA quantities from the tissue samples and exhibited adequate purity (Table 3).

### 3.2. Sequencing and Genotyping Results

Table 4 presents the summary of the results; the number of successfully amplified samples over the number of all the samples tested for a given fragment. The sample was considered successfully amplified if it could also be sequenced (for mt markers) or genotyped using dCAPS protocol (for SNP 2 and 3).

**Figure 2:**
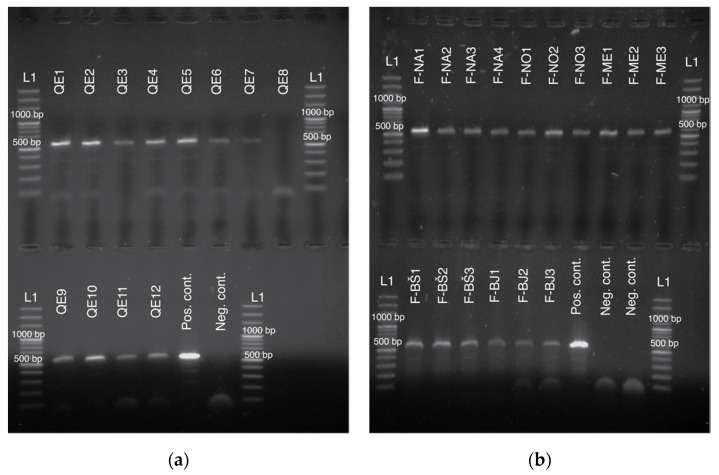
Agarose gel electrophoresis of the amplified fragment for mitochondrial marker tRNA^leu^-COX2. Extracts of DNA from queen cells (**a**) and queens’ feces (**b**). For positive control, one extract from the leg was used. L1 denotes GeneRuler 100 bp Plus DNA ladder; 500 and 1000 bp bands are labeled.

**Figure 3:**
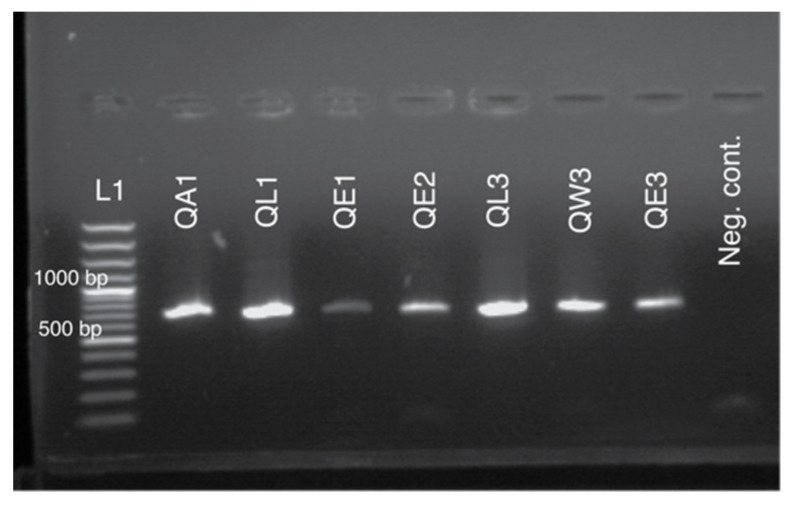
Agarose gel electrophoresis of the amplified fragment for mitochondrial marker cytochrome oxidase I (COI) from various DNA sources: QA—antennae; QL—leg; QE—exuviae; QW—wing clipping. For positive control, one extract from the leg was used. L1 denotes GeneRuler 100 bp Plus DNA ladder; 500 and 1000 bp bands are labeled.

Despite lower concentration and sub-optimal purity of DNA extractions from non-destructive sources, we successfully amplified two mitochondrial DNA fragments (COI and tRNA^leu^-COX2) from all different sources of starting material. Figure 2 and Figure 3 present visualized amplified PCR products for tRNA^leu^-COX2 (Figure 2) and amplified products for COI (Figure 3).

Homology check using BLAST nr/nt nucleotide collection database confirmed that all the successfully obtained sequences for two mitochondrial markers belong to *Apis mellifera* (Supplementary Materials—Table S1). tRNA^leu^-COX2 fragment was successfully amplified in 120 samples. Six different variants were identified, and the sequences are deposited in the Genbank repository under accession numbers MW082028–MW082033.

Universal COI gene region is also a globally recognized barcode [25]. Our results revealed the variation between *A. m. carnica* samples from Slovenia of this mitochondrial region for the first time. The fragment is 661 bp long and was successfully amplified in 89 samples altogether, yielding three variants that we compared with records in the BOLD database and performed BLAST searches (Supplementary Materials—Table S1). The sequences are deposited in the Genbank repository under accession numbers MW082034–MW082036. Since oligonucleotide primers used to amplify COI gene region are conserved among invertebrates, sequencing of this fragment confirmed the identity and excluded the possibility of contamination and/or false-positive results due to amplification of other sources than honeybees.

Phylogenetic analysis using the Bayesian approach placed all the selected mitochondrial sequences among other Western honeybee samples (Supplementary Materials—Figure S1). While this result does not answer specific phylogenetic questions and does not provide any novel insight into phylogenetic relationships, this analysis’s primary purpose is to demonstrate that various non-destructive and semi-destructive samples of honeybees may also be useful for phylogenetic relationships reconstruction.

**Figure 4:**
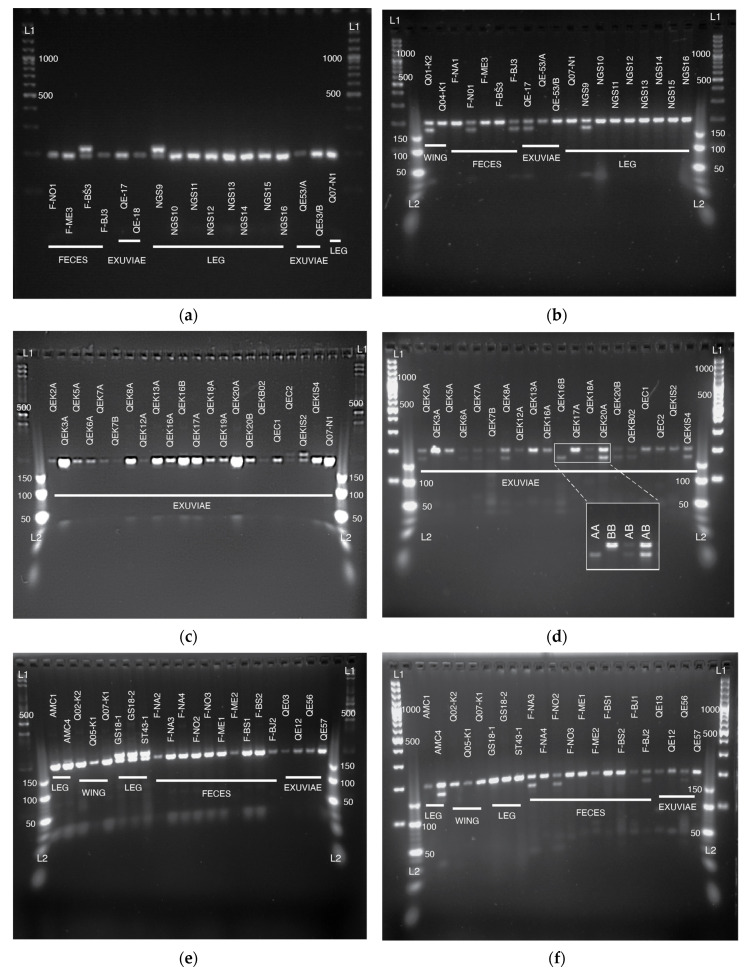
Agarose gel electrophoresis of dCAPS (derived Cleaved Amplified Polymorphic Sequences) restriction of successfully amplified loci (**a**,**c**,**e**) for SNP2 and (**b**,**d**,**f**) for SNP3. There are three possible combinations of cleavage results: both homozygous (AA, BB) and heterozygous (AB). An example is given in inset in (**d**), with three possible outcomes on the gel’s cut-out section. Double bands in any of the figures denote heterozygous state at each SNP. For SNP2, samples exhibiting heterozygosity are: F-BŠ3, NGS9, QEC2, QEKIS2, GS18-1, GS18-2, and ST43-1. For SNP3, samples exhibiting heterozygosity are: Q01-K2, F-NO1, F-BJ3, QE17, NGS9, QEK2A, QEK6A, QEK7A, QEK8A, QE18A, QE20A, QE20B, QEKB02, QEKIS4, AMC4, F-NA3, F-NO2, F-BJ2, and QE56. In (**a**,**b**), the sample names starting with F denote feces samples, sample names starting with QE denote exuviae samples, sample names containing K denote wing clippings, and sample Q07-N1 denotes queen leg. The labels not containing any of the above marks are samples obtained by destructive sampling. L1 denotes GeneRuler 100 bp Plus DNA ladder, and 500 and 1000 bp bands are marked. L2 marks O’RangeRuler 10 bp DNA Ladder, 50, 100, and 150 bp bands are marked.

SNP2 and SNP3 loci each contain a single nucleotide polymorphism linked to varroa sensitive hygiene (Table 2). Amplified SNP2 fragment is 217 bp long. If it contains T at the polymorphic site, it is specifically digested with *Alu*I restriction endonuclease to produce two fragments, 192 and 25 bp in length. The variant with C at the polymorphic site remains undigested. SNP2 locus genotyping success was 81% from exuviae, 94% from feces, 100% from wing-clippings, and 100% from legs (Figure 4a,c,e). Amplified SNP3 fragment is 201 bp long. If it contains C at the polymorphic site, it is specifically digested with *Mnl*I restriction endonuclease to produce two fragments, 172 and 29 bp in length. The variant with T at the polymorphic site remains undigested. SNP3 locus genotyping success was 75% from exuviae, 94% from feces, 100% from wing-clippings, and 100% from legs (Figure 4b,d,f). DNA extracts from antennae were not genotyped. Amplification results for each SNP and additional agarose gel electrophoresis images of restriction are presented in Supplementary Materials—Figures S2–S4.

## 4. Discussion

Molecular methods like classical genotyping and NGS (next-generation sequencing) are invaluable in the plethora of applications, from identifying an individual to determining its pedigree, and are in many cases steadily replacing older morphometric methods. Much like insect collections in natural history museums, collections of various gene sequences were established to serve as a reference to researchers. Both the traditional and molecular methods require a reliable sample, such as the organism itself, its body part, or a fragment of its tissue. Often such sampling—like the traditional insect-pin collection—means the destruction of the individual. In honeybee queens, non-destructive and semi-destructive biological samples, like exuviae, feces, and clipped wings, are desirable sources: they allow genotyping without sacrificing the individual and have very little or even no impact on its survival. Additionally, the samples directly represent the individual of interest’s genotype and are not prone to error due to underrepresentation when sampling the offspring, and thus no subsequent reconstruction is required.

Many different DNA extraction methods from insects have been successfully used previously [7,8,15,17,39]. The method we chose is a commercial kit reported to have a wide collection of uses covering non-standard and non-tissue samples. Since the non-destructive samples such as feces and exuviae are expected to contain degraded DNA of small quantities and polymerase reaction inhibitors, we have chosen this method over the simpler and cheaper Chelex^®^ 100 extraction method [40] to avoid steps where DNA may be disrupted further and to eliminate potential inhibitors of the PCR reaction. For instance, it has been shown that Chelex^®^ 100 extraction method from wing-clippings resulted in much lower PCR amplification than it has been reported in previous studies [15]. The reasons for this may be the degradation of DNA during the extraction or the presence of inhibitors of PCR reaction.

We demonstrated that the extraction of DNA from non-destructive samples might be used in studies of mitochondrial and nuclear DNA. Fragments of mitochondrial DNA could be successfully used for molecular determination of lineages in honey bees, which is traditionally based on tRNA^leu^-COX2 fragment [5]. We confirmed that all the processed samples belong to the C lineage. COI, a protein-coding mitochondrial gene, is a universal barcoding marker [28] used for species determination [41] and was chosen in our study for monitoring the possible contamination of the DNA extracts with foreign DNA. Additionally, COI marker variability was assessed for the first time for Slovenian *A. m. carnica,* and three variants were determined. The success rate varied for both mitochondrial markers (Table 3). Although mitochondrial DNA is inherited maternally and may be satisfactorily determined from the worker honeybees without sacrificing the queen, this is not the case with the detection of polymorphic molecular markers on nuclear loci. SNPs detection could be used for selection of honeybees for desired traits [12,36,42]. At the time being, the commercial operators provide SNP analysis at a relatively high price per sample, and at least fifty samples are required per colony to determine its queen’s genotype. This could be an important reason why the breeding programs do not routinely incorporate such genomic selection yet. Another drawback is that the identified genetic basis of hygienic behavior and other varroa-related traits differ between studies [43]; thus, each population has a specific baseline. For now, only polypeptide markers proved to be suitable for industry-scale breeding, though [44]. For the sake of the proof of concept, we constructed dCAPS markers that enable detection of certain SNP in each sample by taking advantage of specific recognition and cleavage using restriction endonucleases. We used *Taq* polymerase for amplification of SNP containing regions. Since it lacks proofreading mechanisms, a portion of uncuttable fragments in PCR reaction may arise from the replication error. Despite this, standard *Taq* polymerases are used widely in similar studies [45,46,47]. Nevertheless, we advise using high-fidelity polymerases when screening populations to avoid such errors.

Of course, the success of the non-destructive approach is not guaranteed on all occasions and purposes. Obtaining useful genetic information from samples, collected in a non-destructive way, seem to have a wide range of success rate among different insect taxa and for different purposes [14,15,17,21,48]. These results hint that any non-destructive approach should not be considered universal but rather decided upon *after* preliminary screening. For example, using fecal pellets and exuviae for microsatellite analysis in a population study of dragonfly species *Somatochlora hineana*, was not successful [49]. It seems that these two non-destructive sources simply did not provide enough DNA material for those two methods. Our own success rate concerning SNPs for exuviae is comparable: 81% for SNP2 and 75% for SNP3. In another study, the average call rate for SNP obtained from queen exuviae was 83% [12], yet the methodology differed from ours: SNP chip vs. small-scale classic genotyping. Results from feces samples seem to be more promising; the success rate for both selected SNPs was higher, 94%. However, feces as a sample for molecular analyses has some serious limitations: the risk for contamination, presence of PCR inhibitors, and degradation of the DNA and thus difficulties of amplifying long sequences [50]. Feces itself also contains traces of other organisms living as parasites or as symbionts in the gut of honeybee individuals [8,51], which can be important when determining the suitability of a queen to head the colony and breed.

Nevertheless, in our study, feces as a starting material performed better than exuviae. An important technical observation is that the DNA extraction method from feces was more straightforward and contained fewer steps than the extraction of DNA from exuviae. Legs and wing-clippings as a starting material performed better than antennae. Our results show that both, chitin remnants of exuviae in queen cells after queen’s emergence as well as queens’ feces, are acceptable alternatives to semi- and destructive sources useful especially in classical genotyping of an individual without sacrifice. Yellowish mass present at the bottom of the queen cell was not considered a reliable source of queen genotyping since it may contain remnants of royal jelly and represent the genotype of workers in the colony. Furthermore, there were some difficulties with the extraction procedure, as mixing it with the lysis buffers and ethanol produced a viscous solution that clogged the spin column. Our results also indicate that when wing clipping is justifiable (after the queen mates), this may be the source of choice as a starting material. DNA isolates from antennae were at least suitable for further analyses because quantity and quality of extracted DNA were low.

Molecular approaches requiring sampling are also in use in the conservation of other hymenopteran and insect taxa, which are not (eu-)social (e.g., [52,53,54]) and in which sampling by trapping might impact the population. It is also important to note that non-destructive techniques provide little to no stress to the sampled individual. Like in the case of bumblebees, where the individuals were kept at maximum for thirty minutes [23], the honeybee queens in our case were kept for a short period—twenty minutes at most—separately from their retinue in order to defecate. To our knowledge, such an approach did not hamper their later acceptance into the colonies, into which they were placed over the next two days. In wild bees, feces sampling seems to be preferred, at least with regards to the ease of obtaining the sample in cases where the nest is not known to be able to obtain exuviae. In addition, at least in honeybee colonies, obtaining exuviae can be a methodological challenge. If virgin queen emerged in the colony, the worker bees often clean the cell shortly after its emergence [55]. The most reliable way for obtaining samples of exuviae is to use individual cages in the incubator.

It seems that non-destructive sampling methods should be a welcome step in genomic selection or screening from the point of economics. Samples of the queen’s exuviae or feces could be collected before it is put into the mating nuc. To prepare a mini mating nuc, at least 250–300 mL (cca 1000 individuals) [56] of young worker bees are needed. To obtain young worker bees, producing colonies must be weakened, which leads to lower honey production. The rapid analysis would save the costly mating nuc forming and waiting for the first (drone) brood to appear, which usually happens when both there is enough pollen flow and when the colony reaches a certain size [57], normally not in the same season as the new queen is put in the colony. Therefore, a decision could be made early in the queens’ life, whether it is suitable for further breeding and/or performance testing or not. Such an approach could also improve the economics of bee breeding by shortening generation intervals, like proposed in [58], and avoiding performance testing of the queens that do not pose certain genetic traits.

There are also some reports on the use of honeybee products as sources of DNA, which are another promising non-destructive source harboring immense DNA information. For example, using an appropriate set of primers, the entomological origin of honey can be determined to a certain degree, like distinguishing between species or, in some cases, even between some of the subspecies of honeybees [59,60]. While these sources do not seem directly applicable to queen breeding, genotyping of *csd* alleles using NGS technology yielded a diversity of these alleles within the colony [18]. Even if it may be difficult to envision such an approach in practice, it surely has a practical value in research.

## 5. Conclusions

In our work, we demonstrated the usefulness of two types of non-destructive sampling, which might return some useful parameters about queen quality and its pedigree when asked the correct question. Further optimization of the presented methods will incorporate molecular markers into breeding programs for honeybees, raising the possibility of monitoring the genetic background for local adaptations and selection for desired traits. The described methods enable genotyping of selected honeybee queen immediately after emergence, thus shortening the generation interval and minimizing the risk for under-sampling when sampling a queen’s offspring. DNA extracted from non-destructive samples enables a plethora of molecular studies: detection of introgression of different mitochondrial lineages in conservation breeding programs, the utility of diverse molecular markers, as well as phylogenetic and phylogeographic studies. DNA extraction obtained by non-destructive sampling directly from honeybee queens is also a steppingstone for genomic selection in bee breeding, resulting in faster genetic gain and shortening the generation interval.

## Figures and Tables

**Table 1:** Selected single nucleotide polymorphisms (SNPs), IDs, chromosome number and location, oligonucleotide primer pairs, and restriction endonuclease. The derived site is labeled bold. Here presented dCAPS oligonucleotide primer pairs were all constructed in this study. The reference for the SNPs location is [36].

Locus	ID	Chr. Num	Location	dCAPS Oligonucleotide Primer Sequence (Direction 5′ to 3′)	Restriction Endonuclease
SNP2	AMB-00457689	3	10425353	SNP2F: CCGTGTTCCTTCCTCTCTTTCTCAGC SNP2R: CGAGTTCTCGTCCAGGCATC	*Alu*I
SNP3	AMB-00745078	6	1398456	SNP3F: TCAACCTTCTTTCCTTCTTCCT SNP3R: CAAAACCCCATAAACGCCCC	*Mnl*I

**Table 2:** Amplification conditions, complete and restricted fragment length for each SNP-containing-region.

	SNP2	SNP3
Allele	T/C	C/T
annealing T (°C)	55	50
fragment length (bp)	217	201
restricted fragments size (bp)	192/25	172/29

**Table 3:** The summary of the extracted DNA quantity and purity.

Extracted DNA Source	Mean Quantity (ng/μL) (Range)	Mean 260:280 OD Ratio (Range)
Exuviae	2.7 (0.03 to 21.5)	1.62 (0.35 to 2.13)
Feces	4.5 (0.8 to 21.3)	1.45 (1.09 to 1.79)
Wing clippings	0.91 (0.2 to 2.39)	1.19 (1.01 to 1.88)
Legs	38.72 (7.11 to 239)	2.01 (1.72 to 2.18)
Antennae	0.5 (0.12 to 1)	1.35 (1.06 to 1.56)

**Table 4:** A summary of the results. “+” indicates successfully sequenced or genotyped samples; “total” indicates the total number of samples in amplification.

DNA Fragment	Exuviae +/Total	Feces +/Total	Wing-Clippings +/Total	Legs +/Total	Antennae +/Total
tRNA^leu^-COX2	54/95	16/16	6/8	41/41	3/4
COI	38/53	15/16	8/8	26/27	2/4
SNP2	29/36	15/16	5/5	40/40	n.a.
SNP3	27/36	15/16	5/5	39/39	n.a.

## Supplementary Materials

The following are available online at https://www.mdpi.com/2075-4450/11/12/896/s1, Figure S1. Phylogenetic tree for tRNAleu-COX2 marker using Bayesian approach. Figure S2. SNP2 amplification. Figure S3. SNP3 amplification. Figure S4. Agarose gel electrophoresis of SNP2 restriction. Figure S5. Aligned nucleotide sequences of SNP2 containing regions from preliminary set of samples. Figure S6. Aligned nucleotide sequences of SNP3 containing regions from preliminary set of samples. Table S1. Homology of mitochondrial tRNALeu—cox2 and COI fragments: BLAST searches in nr/nt nucleotide collection database.
