# Supplementary material for: Non-Destructive Genotyping of Honeybee Queens to Support Selection and Breeding
Source: Insects. 2020 Dec 21;11(12):896. doi: 10.3390/insects11120896 (PMC7767382; doi:10.3390/insects11120896)
Supplement: Supplementary file 1 [file insects-11-00896-s001.zip › insects-1019199-s/Supplementary_Figures.docx]

|   (**a**) |
| --- |

**Figure S1.** Phylogenetic tree for tRNA^leu^-COX2 marker using Bayesian approach.

Posterior probabilities are presented on nodes. Orange, blue and green colors denote our experimental samples. Black color denotes records obtained from Genbank with appropriate accession numbers.

| 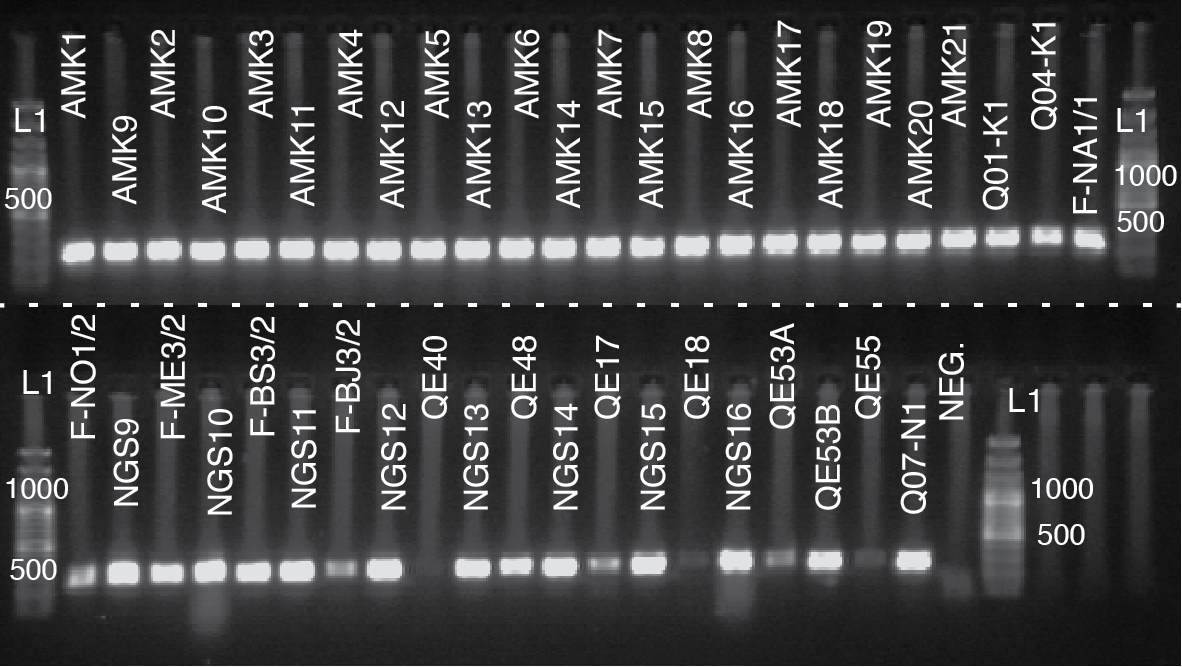  (**a**) |
| --- |

**Figure S2.** SNP2 amplification.

QE – queen exuvia, F – feces, Q07-N1, AMK and other labels – destructive samples (legs). L1 denotes GeneRuler 100 bp Plus DNA ladder. 500 and 1000 bp bands are labeled. Empty area on the electrophoresis gel between the top and bottom lanes was removed. Dotted line marks restitching.

| 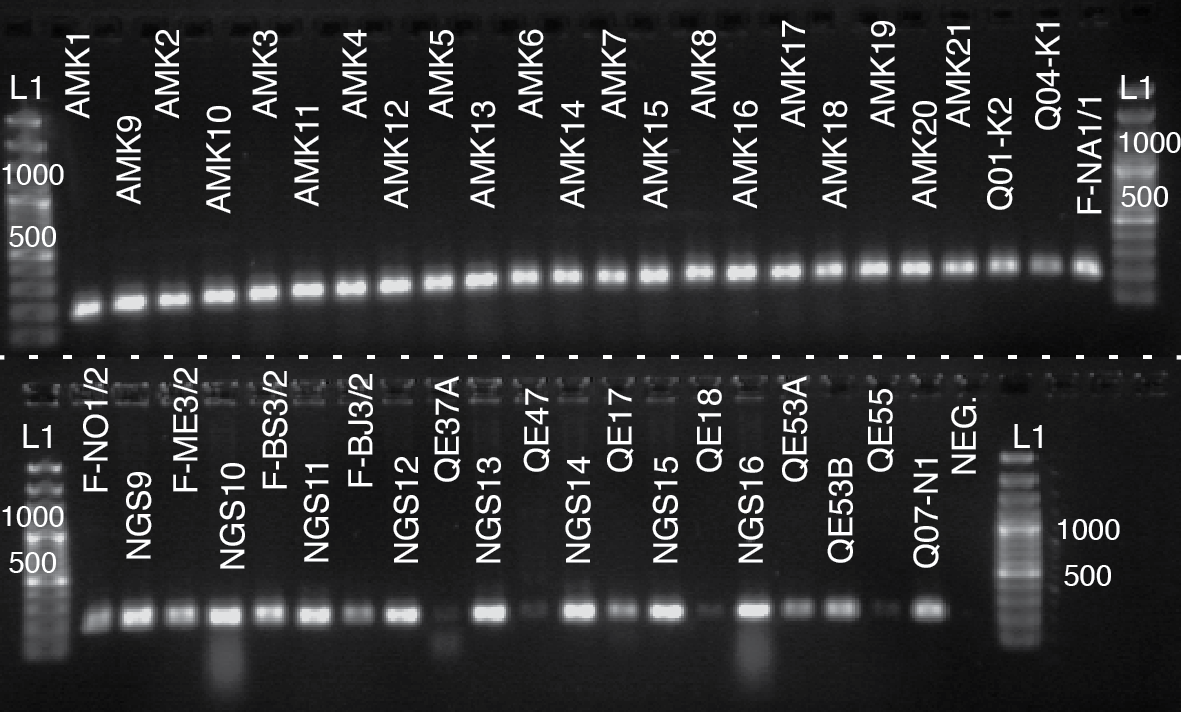  (**a**) |
| --- |

**Figure S3.** SNP3 amplification.

QE – queen exuvia, F – feces, Q07-N1, AMK and other labels – destructive samples (legs). L1 denotes GeneRuler 100 bp Plus DNA ladder. 500 and 1000 bp bands are labeled. Empty area on the electrophoresis gel between the top and bottom lanes was removed. Dotted line marks restitching.


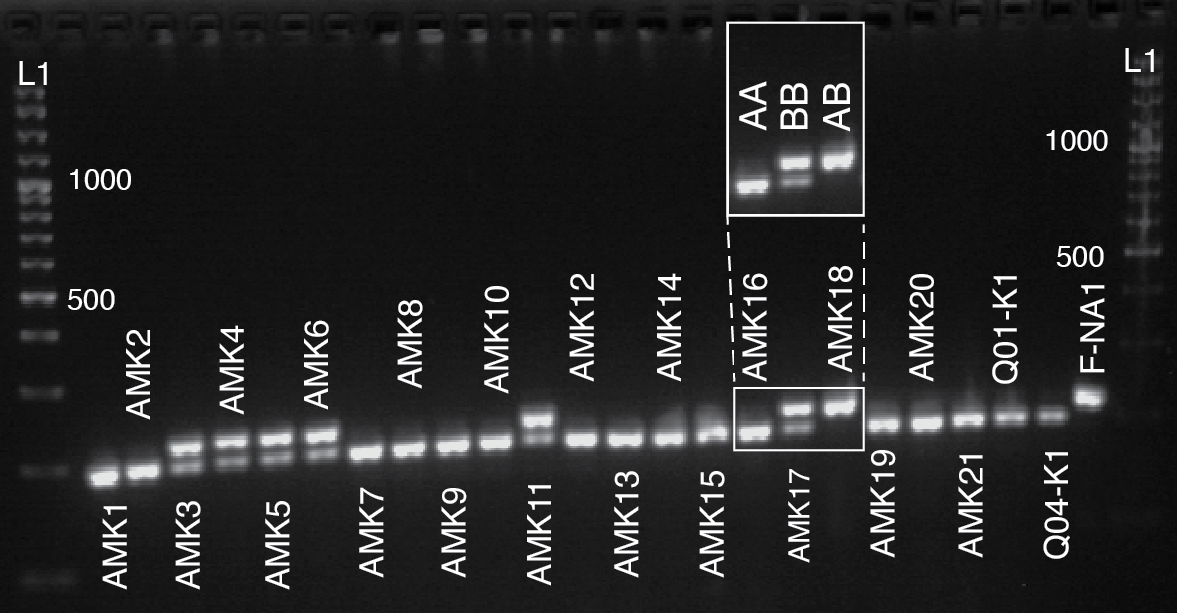


(**a**)

**Figure S4**. Agarose gel electrophoresis of SNP2 restriction.

All three possible outcomes are shown as the result of the dCAPS approach: AA, BB (both homozygous), AB (heterozygous) as shown in *inset*. Q01 and Q04 – queen wing tip, F – feces, AMK mark destructive sampling (legs). L1 denotes GeneRuler 100 bp Plus DNA ladder. 500 and 1000 bp bands are labeled.


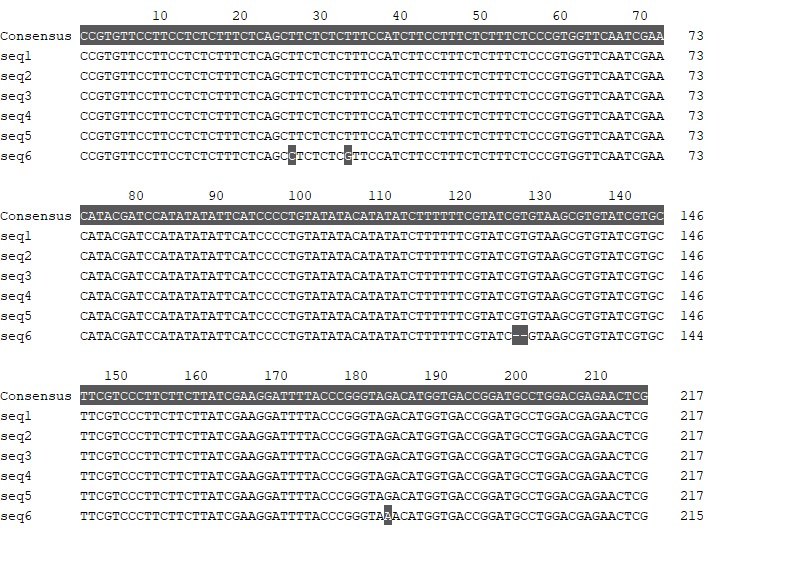
 **Figure S5.** Aligned nucleotide sequences of SNP2 containing regions from preliminary set of samples.


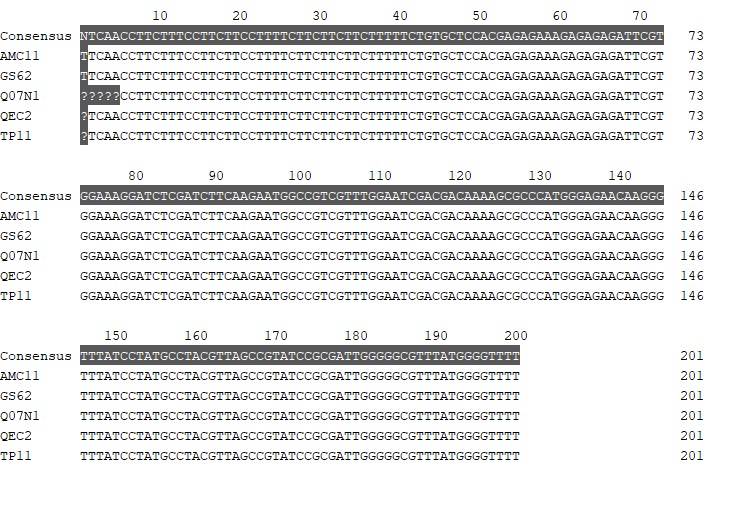
**Figure S6**. Aligned nucleotide sequences of SNP3 containing regions from preliminary set of samples.
